# Discovery of *Encyclometra bungara* (Digenea: Encyclometridae) in a new host (*Enhydris enhydris*) from Thailand and Cambodia through morphological and molecular identification

**DOI:** 10.1017/S0031182023001166

**Published:** 2024-01

**Authors:** Abigail Hui En Chan, Urusa Thaenkham, Akkarin Poodeepiyasawat, Somusa Boonserm, Pakteema Namjad, Panithi Laoungbua, Tanapong Tawan, Ngor Peng Bun, Napat Ratnarathorn, Vachirapong Charoennitiwat

**Affiliations:** 1 Department of Helminthology, Faculty of Tropical Medicine, Mahidol University, Bangkok, Thailand; 2 Department of Biology, Faculty of Science, Mahidol University, Bangkok, Thailand; 3 Snake Farm, Queen Saovabha Memorial Institute, The Thai Red Cross Society, Bangkok, Thailand; 4 Faculty of Fisheries, Royal University of Agriculture, Phnom Penh, Cambodia

**Keywords:** *Encyclometra bungara*, *Enhydris enhydris*, molecular identification, morphology

## Abstract

The genus *Encyclometra* is one of the two genera in family Encyclometridae, known for parasitising the oesophagus, stomach and intestine of snakes. Among *Encyclometra*, the species present are: *Encyclometra colubrimurorum*, *Encyclometra japonica*, *Encyclometra asymmetrica* and *Encyclometra bungara*. Species discrimination within *Encyclometra* has predominantly relied on morphological differences, such as the length of the caeca and the position of the testes. Morphological overlaps exist among these species making species discrimination challenging. Additionally, the use of molecular information has been limited for *Encyclometra*. To determine the *Encyclometra* species infecting *Enhydris enhydris* from Thailand and Cambodia, morphological and molecular identification was conducted. Morphological characters and measurements were obtained from 30 *Encyclometra* adults, and they were compared with previous studies of other *Encyclometra* species. Novel sequences of *E. bungara* were generated using the nuclear 18S and 28S ribosomal RNA genes, and the mitochondrial cytochrome c oxidase subunit 1 gene. Our results revealed that the specimens could be morphologically identified as *E. bungara*, with support from molecular information obtained from the phylogenies of the 3 genetic markers employed. Molecular analysis showed that the *Encyclometra* specimens were distinct from *E. colubrimurorum* and *E. japonica*. Through morphological and molecular identification of the *Encyclometra* specimens found in *E. enhydris* from Thailand and Cambodia, we describe and provide a record of *E. bungara* in a new host and new locality. Additionally, novel molecular sequences were generated, revealing the phylogenetic position of *E. bungara* within the superfamily Gorgoderoidea.

## Graphical abstract

**Figure:**
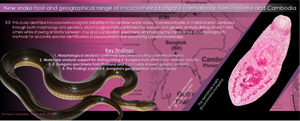

## Introduction

*Encyclometra* Baylis & Cannon, 1924, the genus in the Encyclometridae Mehra, 1931, is classified under the superfamily Gorgoderoidea Loose, 1901. *Encyclometra* is one of the two genera in the family Encyclometridae, where they parasitise the oesophagus, stomach and intestine of snakes (Tkach, 2008; Saito *et al*., 2022). *Encyclometra* is characterised by well-developed oral and ventral suckers, with the oral sucker being smaller or similar in size to the ventral sucker. It is also differentiated from *Encyclobrephus* Sinha, 1949, the other genus in Encyclometridae, by having a tegument without spines (Tkach, 2008).

The species of *Encyclometra* include *Encyclometra colubrimurorum* Rudolphi, 1819, *Encyclometra japonica* Yoshida and Ozaki, 1929, *Encyclometra asymmetrica* Wallace, 1936 and *Encyclometra bungara* Srivastava and Ghosh, 1968 (Rudolphi, 1819; Yoshida and Ozaki, 1929; Wallace, 1936; Yeh, 1958; Srivastava and Ghosh, 1968). The type species *E. colubrimurorum* was first discovered in a grass snake (*Natrix natrix*) in Europe, and it was initially morphologically described by having tandem testes and caeca that are equal in length (Rudolphi, 1819). *Encyclometra japonica* was first discovered in a Japanese striped snake (*Elaphe quadrivirgata*) in Japan and was described as a new species due to the testes positioned diagonally relative to each other (Yoshida and Ozaki, 1929). The third species, *E. asymmetrica*, has been described in various snake species and is characterised by intestinal caeca that are significantly unequal in length (Wallace, 1936). Finally, *E. bungara* was first described in *Bungarus fasciatus* in India (Srivastava and Ghosh, 1968) and has also been documented in southeast Asian countries (Thailand and Lao People's Democratic Republic) in *Xenochrophis piscator* and *Enhydris plumbea* (Scholz and Ditrich, 1991; Wongsawad *et al*., 1991). Species discrimination within *Encyclometra* has primarily relied on morphological differences in the length of the caeca and position of the testes. However, the criteria for species discrimination among the 4 valid species remain unclear. For example, Yeh (1958) provided the key to distinguish species of *Encyclometra* based on differences in caeca length (equal, subequal or very unequal), while Gupta and Mehrotra (1977) amended the key and suggested the testes position can be used for species discrimination (Gupta and Mehrotra, 1977).

Molecular methods for species identification are not widely utilised for *Encyclometra*. To date, there are limited sequences in reference databases, with sequences only available for *E. colubrimurom* and *E. japonica*. Here, in order to identify the *Encyclometra* species found in *Enhydris enhydris* from Thailand and Cambodia, a comparison of morphological characters among the other valid species of *Encyclometra* was performed. Additionally, molecular identification using both nuclear and mitochondrial genetic markers was used to obtain novel sequences for *E. bungara*.

## Materials and methods

### Parasite isolation

A total of 18 rainbow water snakes (*E. enhydris*) were obtained from southern Thailand in Nakhon Si Thammarat and adjacent provinces, north-eastern Thailand in Mahasarakham province and Kampong Chhnang province in Cambodia. The snakes were dissected to examine parasites present in the oesophagus. The adult trematodes were isolated, counted and then preserved in 70% ethanol.

### Morphological analysis

Thirty adult trematodes were selected, stained in acetic carmine and mounted in permount for morphological analysis. Morphological characters were observed and measured using an inverted compound microscope equipped with a camera and software (ZEISS primovert, Germany). These characters included body length, maximum body width, oral and ventral sucker dimensions, testes dimensions and position, intestinal caeca symmetry and egg length and width. The morphological characters and measurements were then compared with those from previous studies on *Encyclometra* (Yeh, 1958; Gupta and Mehrotra, 1977; Scholz and Ditrich, 1991; Wongsawad *et al*., 1991; Saito *et al*., 2022). Morphological drawings of the specimens were performed under a compound light microscope to illustrate the morphological characters.

### Molecular analysis

Four trematodes (2 from Thailand and 2 from Cambodia) were selected and individually placed into 1.7 mL microcentrifuge tubes and washed thoroughly with sterile distilled water. Total genomic DNA was isolated using the DNeasy Blood & Tissue kit (Qiagen, Hilden, Germany) following the manufacturer's recommendations. Polymerase chain reaction (PCR) amplifications of the partial regions of the nuclear 18S rRNA gene (C for: 5′-ATGGCTCATTAAATCAGCTAT – 3′, A rev: 5′ – TGCTTTGAGCACTCAAATTTG – 3′), 28S rRNA gene (Digl2: 5′ – AAGCATATCACTAAGCGG – 3′, 1500R: 5′ – GCTATCCTGAGGGAAACTTCG – 3′) and the mitochondrial cytochrome c oxidase subunit 1 (*COI*) gene (JB3: 5′ – TTTTTTGGGCATCCTGAGGTTTAT – 3′, JB4.5: 5′ – TAAAGAAAGAACATAATGAAAATG – 3′) were performed in a final PCR volume of 30 *μ*L (Bowles *et al*., 1992; Curran *et al*., 2011; Routtu *et al*., 2014). The lengths of the partial regions of the 18S rRNA, 28S rRNA and *COI* genes are 800, 1200 bp and 440 bp, respectively. Each reaction contained 15 *μ*L of 2× i-Taq™ mastermix (iNtRON Biotechnology, Gyeonggi, South Korea), 0.1–0.5 *μ*M of each primer and 1 ng *μ*L^−1^ of DNA. PCR was conducted in a T100™ thermocycler (Bio-Rad, California, The United States of America), and the thermocycling conditions followed the respective publications of the primer sequences. PCR amplicons were visualized on 1% agarose gel stained with SYBR™ Safe (Invitrogen, Massachusetts, The United States of America). DNA products were subsequently sent for Barcode Taq sequencing (Celemics, Seoul, South Korea).

Electropherograms of the partial 18S rRNA, 28S rRNA and *COI* genes were manually checked using Bioedit 7.0 (Hall, 1999). A dataset containing the concatenated 18S + 28S rRNA genes was also analysed by manually merging the partial 18S and 28S rRNA gene sequences. Multiple sequence alignment with reference sequences obtained from the NCBI database (Supplementary Table S1) was performed using ClustalX 2.1 for each genetic marker (Thompson *et al*., 2002). The aligned sequences were checked, and a suitable nucleotide substitution model was selected for maximum-likelihood (ML) phylogenetic analysis. The substitution model selection and ML phylogenetic analysis was carried out in MEGA X (Kumar *et al*., 2018). *Schistosoma mansoni* and *Fasciola hepatica* were used as outgroups to root the phylogenetic trees. The phylogenetic trees were visualized and labelled using FigTree 1.3.1 (Rambaut, 2009). Pairwise inter- and intra-species genetic distances were calculated using *p*-distance as the model for each genetic marker in MEGA X (Kumar *et al*., 2018).

## Results

### Morphological identification

**Figure 1.**
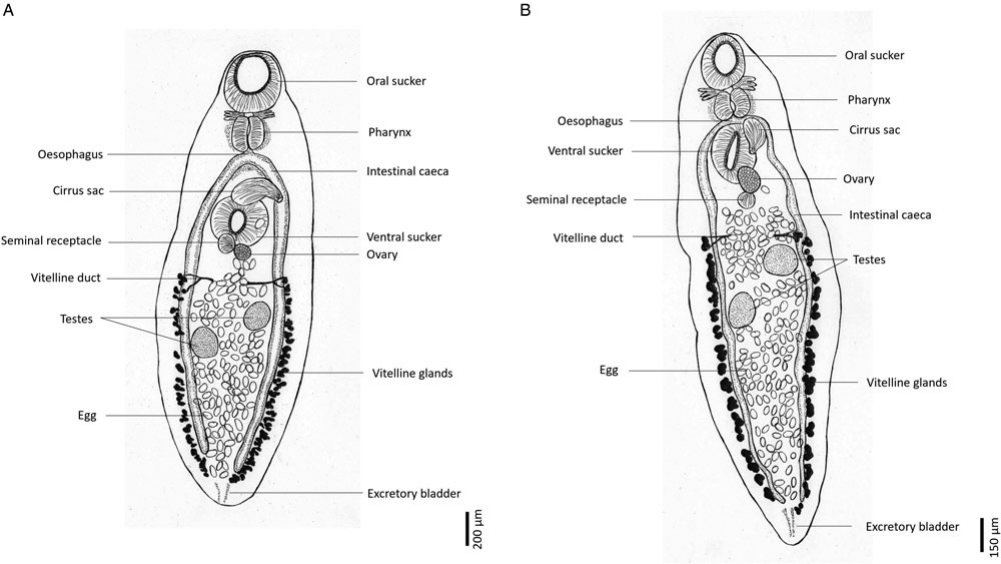
Morphological drawing of adult *Encyclometra bungara* obtained from *Enhydris enhydris* in (a) Thailand and (b) Cambodia (right).

Out of the 18 *E. enhydris* examined, 12 were found to be infected with *Encyclometra*, indicating a prevalence of 66.7%. The infection was in the oesophagus, yielding a total of 103 adult *Encyclometra* specimens. Morphological identification and measurements confirmed that the specimens obtained from both Thailand and Cambodia are *E. bungara*. These diagnostic characters include the diagonally placed testes and equal-length intestinal caeca. The morphological measurements (*n* = 30) obtained from these specimens are presented in Table 1 and Supplementary Table S2. Slight differences in the shape of the posterior end were observed between *E. bungara* specimens from Thailand and Cambodia. The former consistently exhibited a broad posterior end, while the latter consistently displayed a more pointed posterior end. Figure 1 depicts the differences between *E. bungara* obtained from the 2 countries.

**Table 1.** Comparison of *Encyclometra* morphological measurements

Species	*Encyclometra Bungara*			*Encyclometra colubrimurorum*		*Encyclometra japonica*		*Encyclometra asymmetrica*
Reference	This study	Scholz, 1991	Wongsawad, 1998	Yeh, 1958	Gupta, 1977	Yeh, 1958	Saito, 2022	Yeh, 1958
Host	*Enhydris enhydris*	*Enhydris plumbea*	*Xenochrophis piscator*	*Natrix* sp.	*Natrix piscator*	*Natrix* sp., *Enhydris* sp., *Ptyas* sp., *Naja* sp.	*Rhabdophis tigrinus*	*Natrix* sp., *Enhydris* sp., *Ptyas* sp.
Body length	771–2996 (1344)	4320	2360–8840	2900–4900	2769–5596	2000–6000	1497–6198	8000– 10 300
Body width	267–926 (510)	1750	1510–1630	900–1500	1085–1270	700–1600	493–1918	900–1200
Oral sucker	120–263 (192) × 109–257 (185)	600 × 520	290–310 × 180–280	450–550	487–585 × 546–633	360–630	185–434 × 225–517	570–590
Ventral sucker	124–316 (198) × 122–297 (199)	560 × 540	450–510 × 480–520	590–650	614–782 × 643–780	400–760	284–524 × 240–554	510–540
Ratio VS:OS	1.0–1.1: 1	1.1:1	1.1:1	1: 1.1–1.3	1:1.1–1.3	1:1.1–1.2	1:1	1:0.9
Testes	56–192 (97) × 41–170 (89)	270–370 × 180–190	170–320 × 160–190	240–310	107–389 × 136–351	180–500	125–437 × 192–625	470–480
Testes position	Diagonal	Diagonal	Diagonal	Tandem	Tandem or diagonal	Tandem or diagonal	Diagonal	Tandem
Cirrus sac	NA	670	610–800	550–640	390–780	NA	135–527	730
Ovary	NA	140 × 180	100–150	200–240	NA	NA	NA	250–280
Caeca	Equal	Equal	Equal	Equal	Equal	Subequal	Subequal or equal	Very unequal
Egg (length)	60–78 (72) × 32–44 (39)	76–84 × 39–43	75–81 × 31–41	75–84 × 38–46	83–98 × 34–51	74–91 × 42–54	60–101 × 27–63	94–97 × 49–52

Note: measurements are represented as the minimum–maximum (mean) values in micrometres (*μ*m).

VS, ventral sucker; OS, Oral sucker.

The description of *E. bungara* obtained in this study is as follows: whole-body shape flat and lanceolate, posterior end either broad or pointed, cuticle lacking spines. Body length 771–2996 *μ*m, maximum width 267–926 *μ*m. Oral and ventral suckers well-developed, approximately equal in size. Subterminal oral sucker 120–263 *μ*m ×109–257 *μ*m, ventral sucker 124–316 *μ*m×122–297 *μ*m, positioned in anterior quarter to a third of the body. Ratio of ventral sucker to oral sucker 1.1:1. Pharynx distinct, oesophagus very short or absent. Intestinal caeca arise near the pharynx and extend to the posterior end, being equal in length. Curved cirrus sac at anterior border of ventral sucker. Ovary oval, near the posterior border of the ventral sucker, next to seminal vesicle. Two oval or slightly lobed testes, always positioned obliquely in the posterior half of body, 56–192 *μ*m × 41–170 *μ*m. Vitelline glands extend along the side of intestinal caeca, starting from the position between the ovary and testes, and extending to the posterior end of the body. Uterus fully filled, with oval-shaped eggs measuring 60–78 *μ*m×32–44 *μ*m. Excretory bladder Y-shaped.

### Taxonomic summary

#### **Host:** Rainbow water snake, *Enhydris enhydris* (Schneider, 1799) (Squamata: Homalopsidae) **Locality:** Thailand (Southern region: Nakhon Si Thammarat and adjacent provinces; and north-eastern region: Mahasarakham province) and Cambodia (Kampong Chhnang province) **Site of infection:** Oesophagus **Prevalence:** 66.7% (12 out of 18 host infected) **Specimens deposited:** Department of Helminthology, Faculty of Tropical Medicine, Mahidol University

### Molecular and phylogenetic analysis

**Figure 2.**
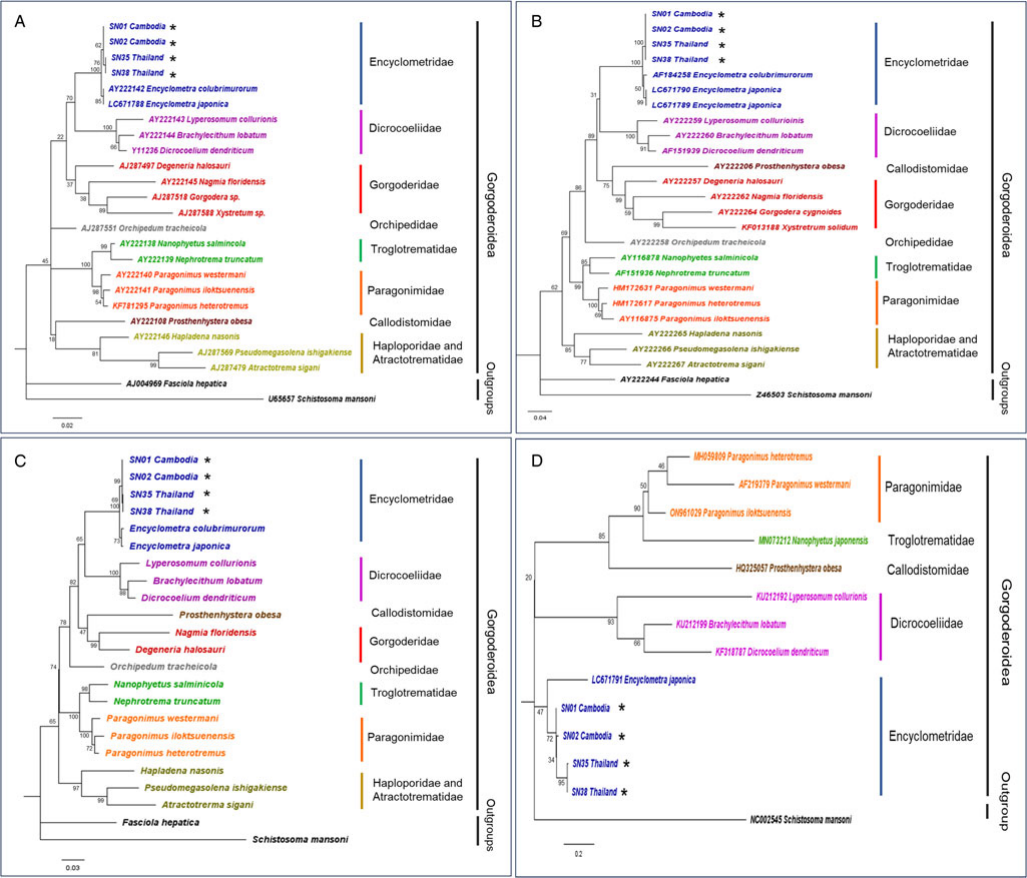
Maximum-likelihood phylogeny using the (a) nuclear 18S rRNA gene (K2 + G + I), (b) nuclear 28S rRNA gene (GTR + G), (c) concatenated nuclear 18S with 28S rRNA genes (GTR + G + I), and (d) mitochondrial *COI* gene (HKY + G). Numbers at nodes indicate bootstrap values. Representative sequences generated from this study are indicated with an ‘*’. The families in the superfamily Gorgoderoidea are colour-coded.

Three molecular genetic markers – nuclear 18S rRNA gene, 28S rRNA gene, mitochondrial *COI* gene and the concatenated 18S + 28S rRNA genes were used for analysis. Firstly, based on the phylogenies obtained, all 3 genetic markers supported that the *E. bungara* specimens obtained from *E. enhydris* in this study were genetically different from *E. colubrimurorum* and *E. japonica* (Fig. 2A–D). Inter-species genetic distances also support that *E. bungara* are distinct from *E. colubrimurorm* and *E. japonica*. The genetic distances between *E. colubrimurorum* or *E. japonica* with *E. bungara* ranged from 0.2–0.4, 1.1–1.2 and 12.0–14.7% using the 18S, 28S and *COI* genes, respectively. Moreover, these genetic distance values obtained were higher than the inter-species genetic distances between *E. colubrimurorum* and *E. japonica*. Table 2 presents the comparison of genetic distances for the 3 genetic markers.

**Table 2.** Inter- and intra-species genetic distances of *Encyclometra*

Species	Genetic marker			
	18S	28S	18S + 28S	*COI*
*E. colubrimurorum vs E. japonica*	0.0	0.8	0.5	–
*E. colubrimurorum vs E. bungara*	0.2–0.4	1.1	0.7	–
*E. japonica vs E. bungara*	0.2–0.4	1.2	0.8	12.0–14.7
*E. bungara* (Thailand) *vs E. bungara* (Cambodia)	0.0– 0.13	0.0	0.0–0.05	0.3–6.3

The values are expressed as the percentage of nucleotide differences.

Secondly, the *E. bungara* specimens from Thailand and Cambodia exhibited genetic similarity, providing evidence that the specimens obtained are the same species. Genetic distances between the specimens using the 18S (0.0–0.13%), 28S (0%) and *COI* (0.3–6.3%) genes were lower than the inter-species genetic distance observed in *Encyclometra*. Additionally, using the 18S rRNA gene, no sequence variation was observed between *E. colubrimurorum* and *E. japonica*. Contrarily, the use of the 28S rRNA gene and the concatenated 18S + 28S rRNA gene showed slight sequence variations of 0.8 and 0.5%, respectively.

Despite low sequence variation using the nuclear genetic markers (Figs 2A–C), the phylogeny obtained was well-resolved, with the monophyly of Encyclometridae and its sister group relationship with the family Dicrocoeliidae. Moreover, phylogenetic relationships within the superfamily Gorgoderoidea (comprising 8 families) were similar with both the nuclear 18S and 28S rRNA genes, except for Callodistomidae and Orchipedidae.

## Discussion

Through morphological and molecular identification of the *Encyclometra* specimens found in *E. enhydris* from Thailand and Cambodia, we provide a record of *E. bungara* in a new host and new locality, along with novel molecular sequences. Secondly, the similarities in morphological characters among the 4 valid species in *Encyclometra* demonstrate the importance of using molecular information for species identification.

*Encyclometra bungara* was previously found in India, as well as in Southeast Asia, including Lao People's Democratic Republic (PDR) and Thailand. Although, to date, this species is not among the *Encyclometra* species that are listed as valid, the morphological descriptions match with previous records of *E. bungara* found in Southeast Asia. Currently, with the results obtained from this study, *E. bungara* can be found infecting 4 snake species. These 4 species are *B. fasciatus*, *X. piscator*, *E. plumbea* and *E. enhydris*. The first record of *E. bungara* in Southeast Asia was reported by Scholz and Ditrich (1991) in the rice paddy snake (*E. plumbea*) from Vientiane Province in Lao PDR (Scholz and Ditrich, 1991). Subsequently, in Thailand, *E. bungara* was found in the checkered keelback snake *X. piscator* during a survey of trematodes in reptiles and amphibians from Doi Suthep-Pui National Park and suburban areas in Chiang Mai Province (Wongsawad *et al*., 1991). Aside from host species, another difference between our specimens and previous studies lies in the site of infection. Our specimens were exclusively found in the oesophagus, whereas previous studies found *E. bungara* in the intestine of hosts. However, *Encyclometra* has also been found to infect various organs of hosts, including the oesophagus, stomach and intestine (Yeh, 1958; Tkach, 2008; Saito *et al*., 2022). Although no molecular information on *E. bungara* was available for comparison, our specimens can be considered conspecific with *E. bungara* due to similar morphological characters such as the diagonally placed testes and equal-length intestinal caeca. Phylogenetic evidence also supported the differentiation of the *Encyclometra* specimens obtained in this study from *E. colubrimurorum* and *E. japonica*, suggesting that the *Encyclometra* specimens obtained are distinct from other *Encyclometra* species.

The rainbow water snake, *E. enhydris*, is endemic in Southeast Asia and has expanded its range to include parts of China, India and Australia (Murphy *et al*., 1999; Karns *et al*., 2000; Lim and D'Rozarlo, 2009; Karns *et al*., 2010*a*). In the southern and central regions of Thailand, previous surveys demonstrated the dominance of *E. enhydris*, comprising more than 80% of the snake species surveyed (Karns *et al*., 2000; Karns *et al*., 2010*b*). *Enhydris enhydris* can thrive in various habitats, including rice paddies, canals and artificial fishponds. Since their diet primarily consists of fish and amphibians (both adults and juveniles), the life cycle of *Encyclometra* can be completed with the rainbow water snake as the final host (Voris and Murphy, 2010). Based on evidence from morphological similarities, geographic localities, host characteristics and molecular data, the specimens obtained in our study expand the known geography and host species of *E. bungara*, thereby affirming the presence of *E. bungara* in semi-aquatic snakes from Southeast Asia.

Comparing the species within the genus *Encyclometra*, morphological similarities are present, and key diagnostic characters such as the intestinal caeca and testes’ position are uninformative for species discrimination. Only *E. asymmetrica* can be successfully differentiated by having unequal intestinal caeca and a slightly larger oral sucker compared to the ventral sucker (Wallace, 1936). Furthermore, there are morphological variations within species that can complicate species identification. For instance, *E. colubrimurorum* was initially described as having tandem testes, but a redescription by Gupta and Mehrotra (1977) showed that *E. colubrimurorum* can present both tandem and diagonally testes (Rudolphi, 1819; Gupta and Mehrotra, 1977; Tkach, 2008). A recent discovery by Saito *et al*. (2022) also revealed morphological variations within *E. japonica*, including both caeca types with equal and subequal lengths (Saito *et al*., 2022). With the overlap of morphological characters among *E. bungara* (presenting diagonal testes and equal caeca lengths), *E. colubrimurorum* (diagonal or tandem testes and equal caeca lengths) and *E. japonica* (diagonal or tandem testes and equal or subequal caeca lengths), the use of molecular data is valuable.

Previous studies have demonstrated the importance of molecular data for species discrimination among trematodes, especially among closely related and morphologically similar species (Nolan and Cribb, 2005; Thaenkham and Waikagul, 2008; Nadler and Pérez-Ponce de León., 2011; Thaenkham *et al*., 2011). Given the presence of morphological variations and overlap of morphological characters within *Encyclometra*, the use of molecular information coupled with morphology is recommended (Saito *et al*., 2022). Furthermore, our results also revealed that selecting appropriate genetic markers is crucial for species discrimination. As the nuclear 18S rRNA gene is highly conserved, this genetic marker may not provide sufficient sequence variation for species discrimination (Blasco-Costa *et al*., 2016; Chan *et al*., 2021). Our results showed no sequence variation between *E. colubrimurorum* and *E. japonica* using the 18S rRNA gene. Similarly, this genetic marker also provided no sequence variation between the sister species of *Paragonimus heterotremus* and *Paragonimus pseudoheterotremus* (Chan *et al*., 2022). However, albeit the low sequence variation of the nuclear rRNA genes, they provide robust phylogenetic inferences at higher taxonomic levels (e.g. family level and above) for Digenea, rendering them suitable markers for molecular systematics (Olson *et al*., 2003; Chan *et al*., 2021).

Molecular analysis for this study was constrained by the limited number of sequences and genetic markers employed for *Encyclometra*. Nevertheless, the sequences generated from the 3 genetic markers in this study can hopefully provide a more comprehensive insight for future studies on *Encyclometra*.

## Conclusion

This study focused on identifying *Encyclometra* species parasitising *E. enhydris* in Thailand and Cambodia using both morphological and molecular methods. Morphological analysis confirmed the specimens as *E. bungara* based on diagnostic traits like diagonally positioned testes and equal-length intestinal caeca. Molecular analysis, employing nuclear and mitochondrial genetic markers, supported differentiation from other *Encyclometra* species, with significant genetic distances. The research also revealed genetic similarity between *E. bungara* specimens from Thailand and Cambodia, affirming their conspecific status. This work underscores the importance of combining molecular data with morphology for accurate species identification within *Encyclometra* and highlights the need for selecting appropriate genetic markers, and extending the knowledge in wildlife parasites.

## Supporting information

Chan et al. supplementary material 1 Chan et al. supplementary material

Chan et al. supplementary material 2 Chan et al. supplementary material

## Data Availability

The data that support the findings of this study are available from the first and corresponding authors upon reasonable request.

## Supplementary material

The supplementary material for this article can be found at https://doi.org/10.1017/S0031182023001166
