# Supplementary material for: Discovery of Encyclometra bungara (Digenea: Encyclometridae) in a new host (Enhydris enhydris) from Thailand and Cambodia through morphological and molecular identification
Source: Parasitology. 2023 Nov 24;151(1):77–83. doi: 10.1017/S0031182023001166 (PMC10941033; doi:10.1017/S0031182023001166)
Supplement: Chan et al. supplementary material 1 — Chan et al. supplementary material [file S0031182023001166sup001.docx]

**Supplementary table S1.** NCBI sequences used in this study

| **Species** | **Genetic marker** | | |
| --- | --- | --- | --- |
|  | **18S** | **28S** | ***COI*** |
| *Encyclometra bungara* (SN01) | OR543302 | OR543306 | OR541662 |
| *Encyclometra bungara* (SN02) | OR543303 | OR543307 | OR541663 |
| *Encyclometra bungara* (SN35) | OR543304 | OR543308 | OR541664 |
| *Encyclometra bungara* (SN38) | OR543305 | OR543309 | OR541665 |
| *Encyclometra colubrimurorum* | AY222142 | AF184258 | - |
| *Encyclometra japonica* | LC671788 | LC671790,  LC671789 | LC671791 |
| *Lyperosomum collurionis* | AY2222143 | AY222259 | KU212192 |
| *Brachylecithum lobatum* | AY222144 | AY222260 | KU212199 |
| *Dicrocoelium dendriticum* | Y11236 | AF151939 | KF318787 |
| *Degeneria halosauri* | AJ287497 | AY222257 | - |
| *Nagmia floridensis* | AY222145 | AY222262 | - |
| *Gorgodera cygnoides* | - | AY222264 | - |
| *Gorgodera* sp. | AJ287518 | - | - |
| *Xystretrum solidum* | - | KF013188 | - |
| *Xystretum* sp. | AJ287588 | NA | - |
| *Orchipedum tracheicola* | AJ287551 | AY222258 | - |
| *Nanophyetus salmincola* | AY222138 | AY116878 | - |
| *Nanophyetus japonensis* | - | - | MN073212 |
| *Nephrotrema truncatum* | AY222139 | AF151936 | - |
| *Paragonimus westermani* | AY222140 | HM172631 | AF219379 |
| *Paragonimus iloksuenensis* | AY222141 | AY116875 | ON961029 |
| *Paagonimus heterotremus* | KF781295 | HM172617 | MH059809 |
| *Prosthenhystera obesa* | AY222108 | AY222206 | HQ325057 |
| *Hapladena nasonis* | AY222146 | AY222265 | - |
| *Pseudomegasolena ishigakiense* | AJ287569 | AY222266 | - |
| *Atractotrema sigani* | AJ287479 | AY222267 | - |
| *Fasciola hepatica* | AJ004969 | AY222244 | - |
| *Schistosoma mansoni* | U65657 | Z46503 | NC002545 |

A dash ‘-‘ indicate sequence not in the phylogenetic tree
